# Phenology of *Spondias tuberosa* Arruda (Anacardiaceae) under different landscape management regimes and a proposal for a rapid phenological diagnosis using local knowledge

**DOI:** 10.1186/1746-4269-9-10

**Published:** 2013-01-31

**Authors:** Ernani MF Lins Neto, Alyson LS Almeida, Nivaldo Peroni, Cibele C Castro, Ulysses P Albuquerque

**Affiliations:** 1 Biology Department, Universidade Federal do Piauí, Campus Professora Cinobelina Elvas, BR 135, km 3, Planalto Horizonte, Piauí, Brazil; 2 Laboratório de Etnobotânica Aplicada, Universidade Federal Rural de Pernambuco (UFRPE), Recife, Brazil; 3 Department of Ecology and Zoology, Universidade Federal de Santa Catarina, Florianópolis, Brazil; 4 Department of Biology, Universidade Federal Rural de Pernambuco, Recife, Brazil

**Keywords:** Phenological calendar, Ethnobotany, Umbuzeiro, Caatinga, Fournier index, Synchrony index, People perception

## Abstract

**Background:**

Studies aimed at investigating the influence of habitat change on species phenology. Studies that investigate people's perceptions of the phenology of certain species still area few; yet this approach is important for effective decision-making for conservation. The aim of this study was to investigate the phenology of *Spondias tuberosa* Arruda (Anacardiaceae), a native species of economic and ecological importance in northeastern Brazil, in five landscape units (LUs) (Mountain, Mountain Base, Pasture, Cultivated Areas and Homegardens) of a Caatinga region in Altinho, Pernambuco, northeastern Brazil. These data could then be compared with local people's perceptions of the species’ phenophases.

**Method:**

Collection of phenological data was carried out monthly from February 2007 to January 2009 and included activity, intensity and synchronization of reproductive and vegetative phenophases. Ethnobotanical data were gathered using a collaborative approach to access local people’s knowledge about the species’ phenological schedule.

**Results:**

There were no significant differences in the intensity of phenophases among LUs, and there was a correspondence between people’s perception of phenophases and the phenological data collected. The data show that the different management practices for LUs did not influence the phenology of the species.

**Conclusion:**

The main conclusion of this study is the use of traditional knowledge as interesting tool for rapid phenological diagnosis. However further studies need to be developed to test this tool in other environments and cultural contexts.

## Background

Habitat change has been widely demonstrated to influence many aspects of plant reproduction, including reproductive success [1,2], outcrossing rates [2] and interactions with animals, such as pollinators and dispersers [3-5]. However, little is known about the effects of habitat change on plant phenology [6], such as an example, reflections of environmental change on the overlap of flowering between areas, an important phenomenon to maintain genetic variability [7,8]. According to Fuchs et al. [2], the density of flowering individuals in a specific region points to the effective number of pollen donors, which can affect the flow of pollen in the population. Recently, Almeida et al. [9] evaluated the influence of different soil management regimes on the reproductive success and pollinator guild populations of *Spondias tuberosa* Arruda, having as one of the main conclusions that human management may have affected some aspects related to the reproduction of *Spondias tuberosa*, especially the effects of habitat alteration on the pollinator guild of this species.

Most studies of phenological responses of plants to habitat change are related either to forest fragmentation in rainforests [10-12] or to climate change [13]. According to some studies, rainfall is among the main factors associated with changes in phenology of species found in arid and semi-arid ecosystems [14,15]. However, other authors argue that the phenology of some species found in dry forests do not simply depend on rainfall, but also, on the water status of the plant [16,17], for example the capacity to store water and nutrients to be used during drought periods [18]. In any case, rainfall is an environmental variable that cannot be neglected in phenological studies conducted in dry forests. Higher insolation and consequent photosynthetic rates of plants located at forest edges seem to promote higher rates of flowering, fruiting [19,20] and leaf flush [21], and higher temperatures tend to increase leaf fall [22]. Similarly, a study performed in fragments of the Atlantic forest in northeastern Brazil recorded both higher reproductive activity and greater intensity of phenophases at the forest edge as compared to the forest interior [12]. However, this pattern may not occur for some species (as pointed out by Laurance et al. [6] for Amazonian species) and may also depend on the time since edge creation [7]. Other aspects to be considered are the effects of different forms of land management on the phenology of some species [23]. Flowering and fruiting of some species may suffer strong influence of human management. Otero-Arnaiz et al. [23], for example, found that *Polaskia chichipe* individuals cultivated had presented a higher intensity of fruiting compared to wild individuals of the same species. As for flowering, *Stenocereus stellatus* individuals had presented a higher number of flowers in anthesis than the ones presented by wild area individuals [24].

An important approach for studying plant phenology involves the use of local people’s knowledge of phenological events. For centuries, human populations have been selecting and managing plants to meet their basic needs and accumulating knowledge about the plants’ biology, ecology and phenology [25-28]. Thus, access to representation of people about the ecosystem enables the understanding of processes of environmental change, such transformation of the landscape. This information may be very useful for rapid diagnostics because the determination of phenological patterns requires long monitoring periods [29]. Thus, ethnobotanical studies related to plant phenology may contribute to successful management strategies for plant resources, especially for prominent species such as *Spondias tuberosa* Arruda (Anacardiaceae).

*Spondias tuberosa* (locally known as umbuzeiro) is a native fruit tree that occurs in northeastern and part of southeastern Brazil [30] in areas of dry forests called Caatinga [31]. *Spondias tuberosa* is an andromonoecious species that is pollinated by a wide range of insects [9,32,33] and dispersed by vertebrates [34]. This species represents an important resource for pollinators and dispersers because it flowers and fruits during the dry season. Furthermore, its fruits are an important source of nutrition and represent an alternative income source for people during the dry season. As a result, *S. tuberosa* is widely known and managed in the semi-arid region of northeastern Brazil and is also considered a "sacred plant" [35-38]. The umbuzeiro is rarely cut down and may be found in both conserved and cultivated areas and even in Homegardens such as the backyards of houses [38]. Some studies have investigated differences in the reproductive biology of *S. tuberosa* in areas with different management regimes in the Caatinga [32,33], but there are no studies related to the influence of different management regimes on its phenology, or on the local people’s perceptions of this species’ phenophases.

In the city of Altinho, Pernambuco state, there is a rural community called Carão. Carão is located in a Caatinga area with relatively well-preserved native vegetation, along with areas used for pasture, crops and Homegardens. Individuals of *S. tuberosa* are found in all these habitats [38]. The main questions of this study were: a) Are there differences in the vegetative and reproductive phenology of *S. tuberosa* in areas under different management regimes? b) Are local people’s perceptions of phenophases of *S. tuberosa* similar to the actual patterns observed for the species?

Our hypotheses are a) There are few phenological differences among individuals of *S. tuberosa* located in areas under different management regimes because they are very close to each other [39], especially the flowering and fruiting. Moreover, because the Caatinga is a much more open vegetation type than rainforests, there is likely no noticeable edge effect [40] and this further decreases the likelihood of finding differences in plant phenology. b) The local people‘s perceptions of *S. tuberosa* phenology tends to be in agreement with the phenological data obtained in the field, as this is a widely known and used species. This similarity tends to be higher for the fruiting phenology, since the fruits are the main product used in the region [28,38,41]. These assumptions are held on the following premises: Carão community people maintain a close relationship with the resource, evidenced in the highlighted role that this species has within the community [38,42], the intensity of flowering and fruiting observed in a study on the reproductive success of the species, developed in the same area of the present study, had found similarities between the landscape units as for this aspect [9] and a study conducted with species in incipient state of domestication in the semi-arid region of Mexico revealed that the phenology of this species did not vary in relation to management regimes to which these populations are subjected [43]. Almeida et al [9] found no significant differences in the reproductive success of individuals of *S. tuberousa* in the landscape units, justified here more general analysis of the activity and intensity of phenophases the species, thereby allowing emphasize local perception of phenological pattern of *S. tuberosa*.

## Methods

### Study area

This study was conducted in an area of Caatinga [9,38,42,44] with deciduous and sub-deciduous tree species near a rural community called “Carão” in Altinho city, Pernambuco state, Brazil. The city is located in the Borborema highland, with altitudes ranging from 650 to 1000 meters and generally medium to high soil fertility. The climate is classified as semi-arid hot; the rainy season takes place from February to August. In this context, the influence of different landscape management regimes on the phenology of *S. tuberosa* was evaluated in five landscape units (LUs) in the region, namely: Mountain (area with vegetation in regeneration for nearly 50 years – 628m of altitude above sea level), Mountain Base (area of vegetation in regeneration for nearly 15 years – 498 m of altitude above sea level), Pastures (areas of native grassland - 486m of altitude above sea level), Cultivated Areas (cultivated areas of maize and beans- 469 m of altitude above sea level), and Homegardens (backyards - 463 m of altitude above sea level). The choice of these landscape units was based on the study conducted by Lins Neto et al. [45] in which it was determined the history of land use, as well as the landscape units, recognized by the main informants of the place, as well as the study of Almeida et al. [9], which evaluated the fruit and flower production, pollinator guild and frequency of floral visitors of *Spondias tuberosa* individuals submitted to different soil management regimes. It is noteworthy that both works mentioned above were conducted in the same area of the present study. For more information on historical land-use, the reader is referred to the study of Lins Neto et al. [45]. Yet in the latter, a complete analysis of soil fertility has indicated that areas of native pasture and gardens presented high fertility soils [45].

Social and cultural aspects of Carão are available in Araújo et al. [44], Almeida et al. [9] and Lins Neto et al. [38]. However, some information relevant to the present study should be mentioned.

There are currently 189 people living in 61 houses in Carão. The main economic activity is agro-pastoralism with mainly subsistence agriculture (especially corn and bean monocultures and cattle and goat farming). Near Carão there is a hill, locally known as the Serra (mountain) covered almost entirely with native vegetation that has been in a process of regeneration for nearly 50 years [28]. The top of the hill is flat and there are fields of corn, beans and cassava, which are important for the region’s food supply. At the base of the hill is a transitional area between the hill and flatter areas, locally known as the Baixio (lowlands), where some houses are established, most of which are organized into a village. There are also shops, Catholic and Protestant churches and an elementary school. In the flat areas, there are areas of native grasslands and monocultures close to the houses. Other areas of constant activity are the backyards of houses, where residents usually maintain animal and plant (wild and cultivated) cultures.

### Phenology of *Spondias tuberosa* Arruda

For evaluated the pattern phenological of *S. tuberosa* evaluated the activity, intensity and synchronization of reproductive and vegetative phenophases, as well as its relationship with rainfall, in each LU, ten individuals of this specie were randomly selected from each population (totaling 50 individuals) to be included in monthly monitoring between February 2007 and January 2009. The phenophases considered were flowering (flower buds plus flowers at anthesis), fruiting (immature plus mature fruits), leaf flush and leaf fall. The activity was determined by recording the presence or absence of each phenophase. Estimated the percentage of monthly variation of phenological changes in each individual, and was employed the method proposed by Fournier [46] to evaluate the intensity of each phenophase. This method consists of apply a semi-quantitative interval scale with five categories (0–4) and intervals of 25% between each category for each phenophase considered, and calculate the intensity index. The Fournier index per month for each LUs for each phenophase, ranges from 0 to 100%.

To evaluate variation in the synchronization of phenological phases among individuals of the same LU, and among LUs, a flowering synchrony index was applied [47,48]. The synchronization of each phenophase (Xi) was measured separately for ten individuals of each landscape unit by summing the number of months where there was an overlap of the phenophase between a focal individual and the rest of the sample. This synchronization was calculated using the following formula: [Xi = Σij/(N-1) fi], where Σij is the sum of the number of months in which individuals i and j showed a synchrony in one phenophase (with i being different from j); fi is the number of months in which individual i exhibited a particular phenophase and N is the total number of individuals in the sample. The synchrony index of the species Z is calculated by the arithmetic mean of Xi, as follows: Z = ΣXi/N. This index ranges from 0 (no synchrony) to 1 (perfect synchrony) [47,48].

Rainfall is a relevant variable in the case of the Caatinga, therefore this was correlated with the intensity of phenophases. The rainfall data were obtained from the monitoring carried out daily by an experimental basis the Instituto Agronômico de Pernambucano (IPA), which is set at about 13 km from the study area.

It is worth mentioning that the approach serves the purpose of phenological general characterization of phenophases of *Spondias tuberosa* Arruda under different conditions of soil management in order to establish a solid connection with the local perception of those phenophases.

### Local people’s perceptions of phenophases

A collaborative approach, characterized by the sharing of experiences between the researcher and the residents, was used to assess the perceptions of Carão residents regarding the phenophases of *S. tuberosa*. This approach valued the knowledge of local people and resulted in determination of the most appropriate strategies to improve planning and actions [49]. This study is part of the research project “Traditional knowledge, phenology and morphological and genetic variability in populations of *Spondias tuberosa* Arruda (Anacardiaceae) in semi-arid Northeast” that was approved by the Ethics Committee on Research Involving Humans of the Centro de Ciências da Saúde, Universidade Federal de Pernambuco (registry number 401/08). Despite the phenological studies had begun in 2007, the only ethnobotanical approach was developed in 2009, after the aproval of the ethics committee. Everybody that chose to participate in the study sign a Free Consent and Understanding Agreement.

**Figure 1:**
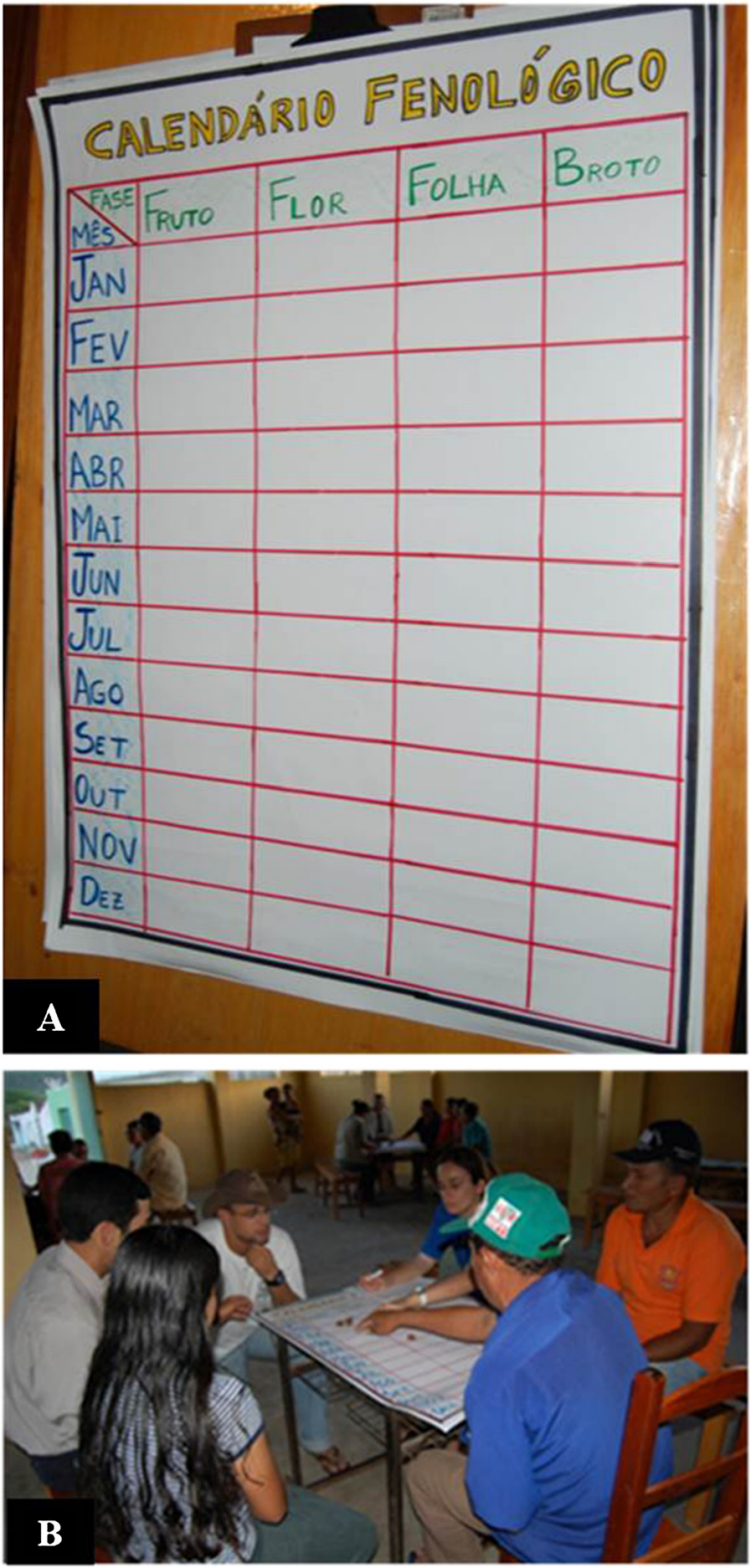
Phenological calendar (A) and group (B) carried out with people form Carao, Altino country Pernamburo, northeastern Brazil.

The participative method used in this study is consistent with an approach called “Score Exercises” that is commonly used to determine the relative importance of the environment and/or resources; the approach consists of local people quantifying the importance of a given resource [49,50]. The method was adapted for this study with the development of a table called the “Phenological Calendar” with the phenophases as the column labels and months as the row labels. All those people (112 informants) known from previous ethnobotanical studies in this community [38] who have mentioned knowledge and/or use of *S. tuberosa* were invited to participate in the activity (70 people). Only 26 people were able to participate and they were randomly assigned to four groups. Despite this low number of respondents, it is emphasized that the vast majority of informants (10 people) of the community when it comes to knowledge and use of umbuzeiro, identified in a previous study [38], participated in this activity. Thus, four groups were formed (two groups of seven and two to six members) (Figure 1). The formation of four groups was made strictly for reasons of management activity, as well as a good distribution of participants. This activity was conducted at the end of March 2009, a period coinciding with the peak of the harvest of umbuzeiro. It is noteworthy that previous studies demonstrate the reliability of the knowledge people have about the plants of the region [42,44], especially umbuzeiro [38,45], a prominent species within the community if compared with other food plants [44].

Each group was coordinated by two members of the research team, the discussion mediator and the person who reported the consensus of the group for each phenophase. The intensity of each phenophase per month was measured on a scale of 0 to 10 (100%), determined by the group’s consensus. The score was determined by the number of “seeds of bean” (the seed number varied from 0, absence of phenophase, 10, maximum intensity, 100%, phenophase of the month) placed in each table cell, (each cell corresponded to one month) (Figure 1). For this analysis, two main habitat types were considered: native vegetation, which comprises the LUs Mountain Base and Mountain, and managed areas, represented by Pasture, Cultivated Areas and Homegardens. These two groups were necessary because most people stated that there is no distinction of phenophases within the managed areas and within native vegetation areas. Similarly, phenological data were grouped into native vegetation and managed areas for comparison with the information on the “Phenological Calendar”.

### Data analysis

To the phenological characterization of the species in the different areas of landscape management, we tested the variation in intensity among LUs, within in year; between years, within LUs; and differences in mean intensity (across two years) among LUs were used a one-way ANOVA (significance level of 5%) with a posterior test of Tukey. Concerning the data of proportion, it was necessary its change into angular values (arcoseno√x/100), and only then apply the ANOVA test. A Spearman’s rank correlation coefficient was used to test the correlation between monthly rainfall and the intensity of phenophases in each landscape unit. The Bioestat 5.0 [51] computer program was used for all analyses.

To test the variation between the perceived intensity of phenophases obtained by "phenological calendar" activity with the intensity measured over two years initially, the perception data were multiplied by 10, since the scale used for phenological timing activity was from 0 to 10. Then both perceived and monitored intensity during two years were transformed to angular values (√ arcoseno x/100) and subsequently tested the variation of the data by ANOVA (one way) with a posteriori Tukey test at 5% probability. The Bioestat 5.0 [51] computer program was used for all analyses. Differences in the perception of intensity of phenophases were also verify using one way ANOVA.

## Results

### Flowering

**Figure 2:**
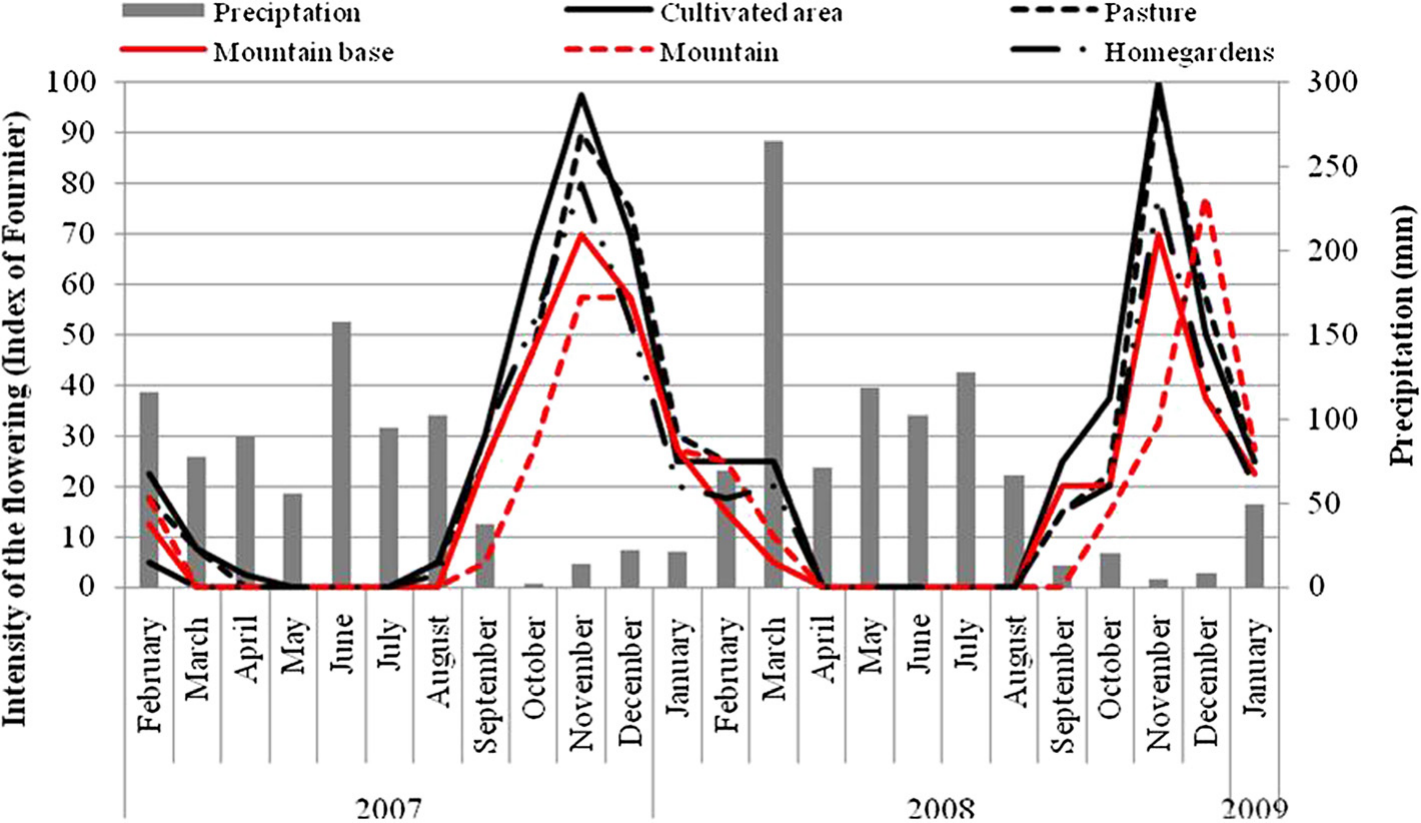
**Intensity of Flowering of *Spondias tuberosa *Arruda (Anacardiaceae) and monthly rainfall curve during February 2007 and January 2009 for the five landscape units in the city of Altinho, Pernambuco, northeastern Brazil.**

During the two years of the study, flowering started in September and ended in April (with a peak in November) in all LUs, (Figure 2). There were variations in the intensity of flowering among landscape units (Figure 2) but they were not significant (first year: F = 0.30; P > 0.05, second year: F = 0.27; P > 0.05). There were no significant differences in the intensity of phenophases in the same landscape unit between the monitoring years (Mountain: F = 0.002; P > 0.05; Mountain Base: F = 0.19; P > 0.05; Pasture: F = 0.04; P > 0.05; Cultivated Areas: F = 0.67; P > 0.05 and Homegardens: F = 0.06; P > 0.05) or in the mean intensity of the two years among landscape units (F = 0.29; P > 0.05). There were no differences in mean intensity between Native vegetation and Managed areas. There was a strong negative correlation between the intensity of flowering and rainfall (Figure 2) in all areas, both in the first (Mountain: *rs* = - 0.71; Mountain Base: *rs* = - 0.77; Pasture: *rs* = - 0.75; Cultivated Areas: *rs* = - 0.74 and Homegardens: *rs* = - 0.72; P <0.01 for all tests) and second year (Mountain: *rs* = - 0.59; Mountain Base: *rs* = - 0.80; Pasture: *rs* = - 0.58; Cultivated Areas: *rs* = - 0.73 and Homegardens: *rs* = - 0.62, P <0.01 for all tests).

### Fruiting

**Figure 3:**
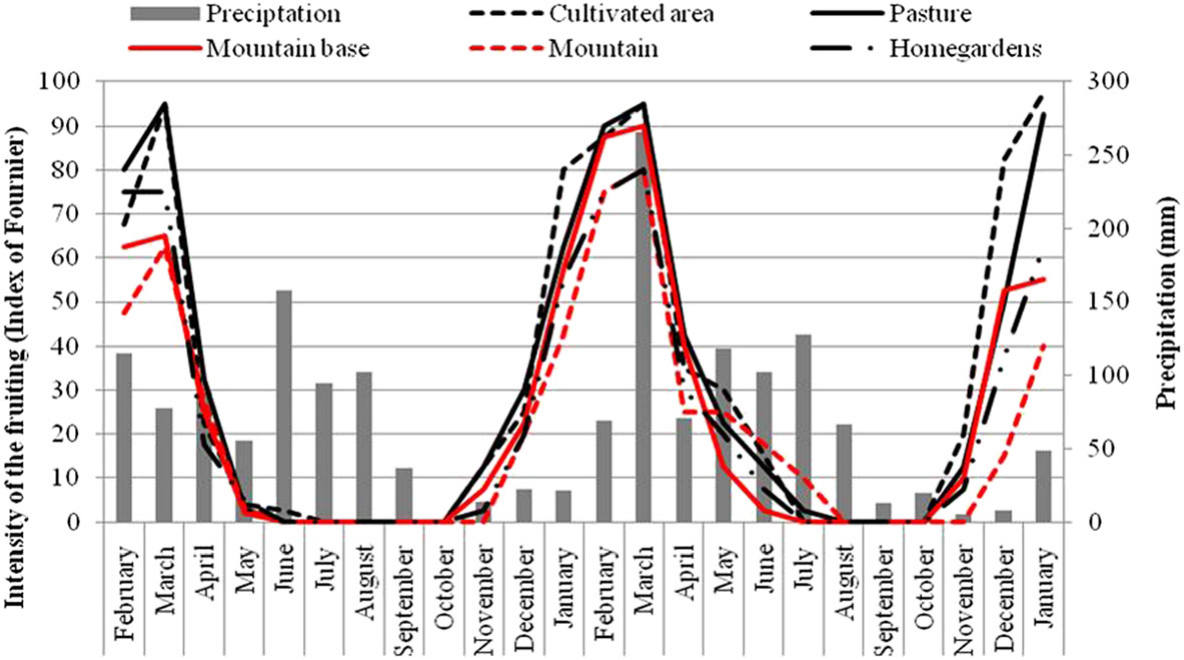
**Intensity of Fruiting of *Spondias tuberosa *Arruda (Anacardiaceae) and monthly rainfall curve during February 2007 and January 2009 for the five landscape units in the city of Altinho, Pernambuco, northeastern Brazil.**

The fruiting period was also similar among LUs, occurring from November to June in the Mountain Base, Cultivated Areas and Pasture, and extending to July in the Mountain and Homegardens. The fruiting peak occurred in March in all LUs. As observed for flowering, there were no significant differences in the intensity of fruiting among individuals in the LUs, either in the first (F = 0.20; P > 0.05) or second year (F = 0.36; P > 0.05, Figure 3), or between the two years (Mountain: F = 0.45; Mountain Base: F = 0.51; Pasture: F = 0.34; Cultivated Areas: F = 0.69 and Homegardens: F = 0.22; P > 0.05 for all tests). The mean intensity of fruiting within the two years did not vary significantly (F = 0.28; P > 0.05, Figure 3). There also significant differences (F = 0.25, P > 0.05) in the intensity of phenophases between native vegetation and managed areas.

Unlike the results for the flowering period, there was no correlation between rainfall and fruiting intensity in the LUs during the first year (Mountain: *rs* = 0.07; Mountain Base: *rs* = - 0.05; Pasture: *rs* = - 0.2005; Cultivated Areas: *rs* = - 0.05 and Homegardens: *rs* = - 0.03; P > 0.05 for all tests; Figure 3). In the second year, only the fruiting individuals of the Mountain were significantly correlated with rainfall (*rs* = 0.58; P < 0.05).

### Leaf flush

**Figure 4:**
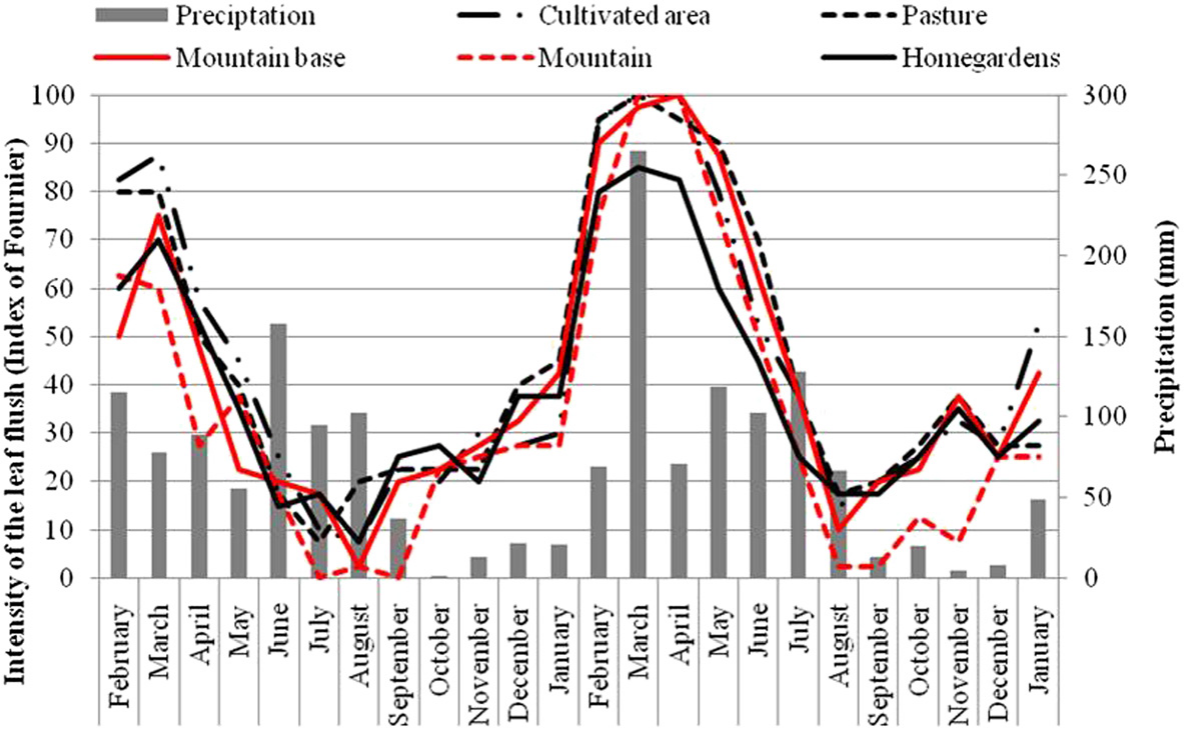
**Intensity of Leaf flush of *Spondias tuberosa *Arruda (Anacardiaceae) and monthly rainfall curve between February 2007 and January 2009 in five landscape units in the city of Altinho, Pernambuco, northeastern Brazil.**

In the two years of monitoring, leaf flush occurred continuously in all landscape units with peak intensity in March (Figure 4). In the first year and in the second year, there were no significant differences in the intensity of this phenophase among LUs (F=0.55; P > 0.05, to fist year; F = 0.36; P > 0.05, to second year) (Figure 4). As observed for other phenophases, there was no significant variation in the intensity of leaf flush in the same LU between years (Mountain: F = 1.68, Mountain Base: F = 3.61, Pasture: F = 1.95; Cultivated Areas: F = 1.78 and Homegardens: F = 1.25; P > 0.05 for all tests) (Figure 4). The mean intensity of leaf flush between the two years did not vary significantly among landscape units (F = 0.43; P > 0.05).

There was no correlation between the intensity of leaf flush and rainfall in the first year (Mountain: *rs* = - 0.02; Mountain Base: *rs* = - 0.16; Pasture: *rs* = - 0.11; Cultivated Areas: rs = - 0.03 and Homegardens: *rs* = - 0.14; P > 0.05 for all tests; Figure. 4). However, in the second year there was a positive correlation in all LUs (Mountain: *rs* = 0.69; Mountain Base: *rs* = 0.60; Pasture: *rs* = 0.64 and Cultivated Areas: *rs* = 0.64; P < 0.05 for all tests), except in Homegardens (*rs* = 0.53; P > 0.05).

### Leaf fall

**Figure 5:**
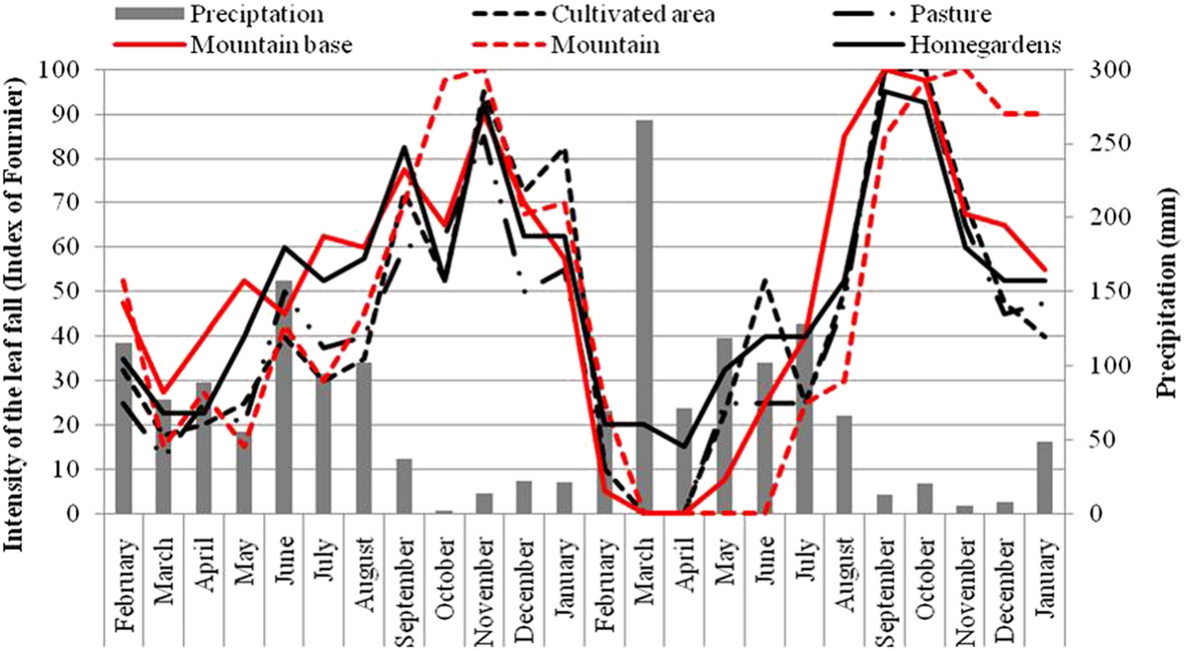
**Intensity of Leaf fall of *Spondias tuberosa *Arruda (Anacardiaceae) and monthly rainfall curve between February 2007 and January 2009 in five landscape units in the city of Altinho, Pernambuco, northeastern Brazil.**

Leaf fall occurred throughout the year in Homegardens and from May to February in Cultivated Areas, Pasture and the Mountain Base. In the Mountain, leaf fall occurred from July to February with peaks in November 2007 and September 2008. As for other phenophases, the intensity of leaf fall did not vary significantly among LUs in either the first (F = 0.67; P > 0.05) or second years (F = 0.06; P > 0.05) or within the same LU (Mountain: F = 0.24; Mountain Base: F = 1.04; Pasture: F = 0.06; Cultivated Areas: F = 0.14 and Homegardens: F = 0.36; P > 0.05 for all tests) (Figure 5). The mean intensity of leaf fall also did not vary significantly among LUs (F = 0.24; P > 0.05).

There was a strong negative correlation between leaf fall and precipitation in the first year for individuals from areas of Pasture (*rs* = - 0.60; P < 0.05), Mountain Base (*rs* = - 0.60; P < 0.05) and Mountain (*rs* = - 0.61; P < 0.05; Figure 4B) and in the second year for all individuals in the LUs Mountain (*rs* = 0.87), Mountain Base (*rs* = - 0.74), Pasture (*rs* = - 0.77), Cultivated Areas (*rs* = - 0.68) and Homegardens (*rs* = - 0.76; P < 0.05 for all tests; Figure 4B).

### Synchrony

Concerning the synchrony, during the two years of the study, it was observed that S. tuberosa individuals were quite synchronous in relation to flowering, fruiting, leaf fall and leaf flush within the LUs and between LUs. Values were very close to perfect synchrony,phenophases overlap occurring phenophases overlap in each of these landscape unit (Table 1).

**Table 1:** **Values for synchrony index of flowering, fruiting, leaf flush and leaf fall of *Spondias tuberosa *Arruda (Anacardiaceae) between February 2007 and January 2009 in five landscape units in the city of Altinho, Pernambuco, northeastern Brazil**

	**Flowering**		**Fruiting**		**Leaf flush**		**Leaf fall**	
	**Year 1**	**Year 2**	**Year 1**	**Year 2**	**Year 1**	**Year 2**	**Year 1**	**Year 2**
Mountain	0.918	0.904	0.899	0.888	0.948	0.919	0.945	1,000
Mountain Base	0.893	0.834	0.868	0.899	0.902	0.934	0.960	0.930
Pasture	0.897	0.834	0.868	0.901	0.879	0.966	0.913	0.973
Cultivated Areas	0.884	1.000	0.866	0.941	0.894	0.946	0.900	0.963
Homegardens	0.940	0.950	0.886	0.822	0.918	0.959	0.921	0.970

### Perception of phenophases of *Spondias tuberosa* Arruda

**Figure 6:**
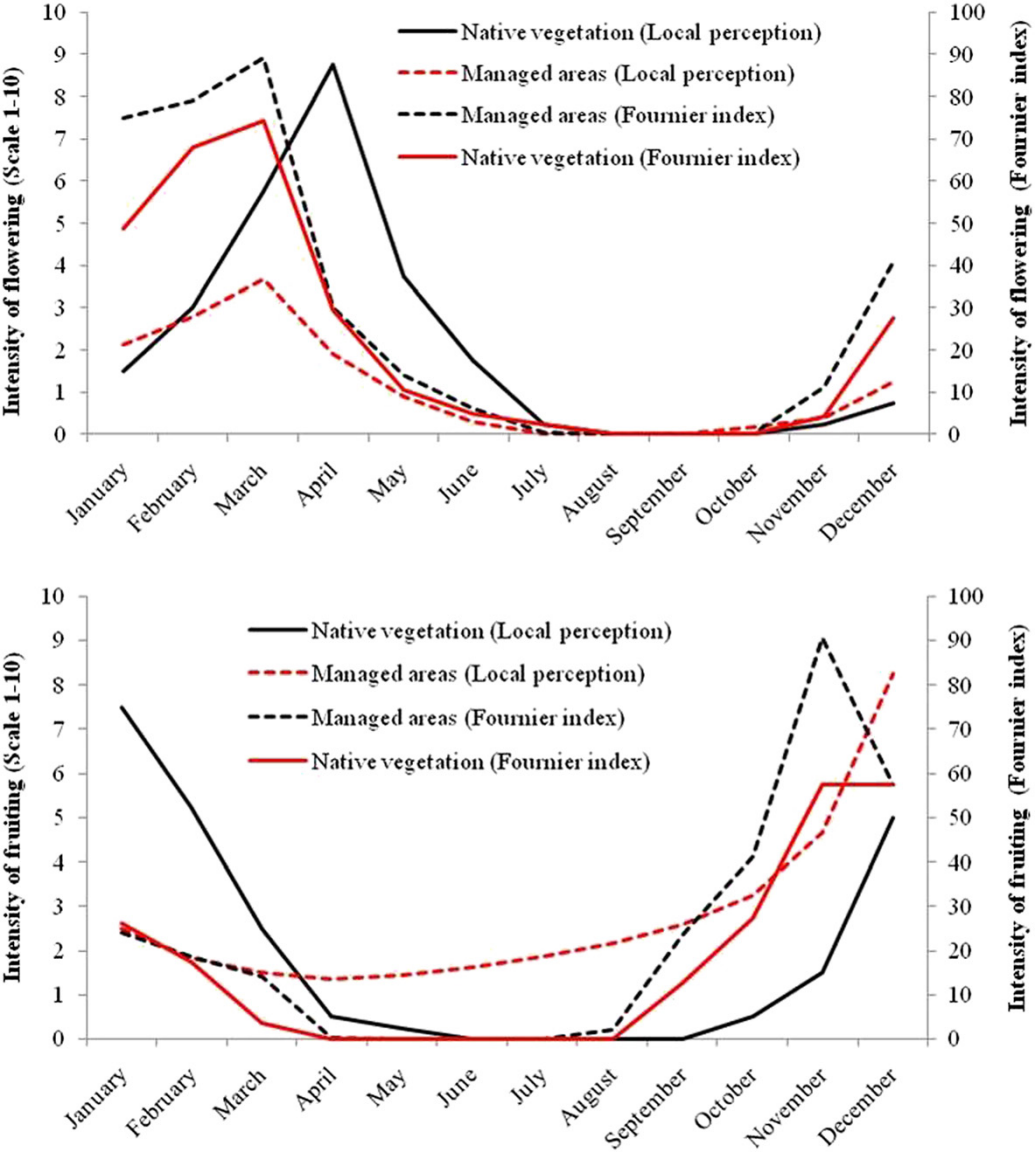
**Comparison of the intensity of flowering and fruiting of *Spondias tuberosa *Arruda in managed areas (Cultivated Areas, Pasture and Homegardens) and native vegetation (Mountain and Mountain Base) with the intensity of phenophases obtained in the collaborative activity "Phenological Calendar" by members of the Carão community, city of Altinho, Pernambuco, northeastern Brazil.**

**Figure 7:**
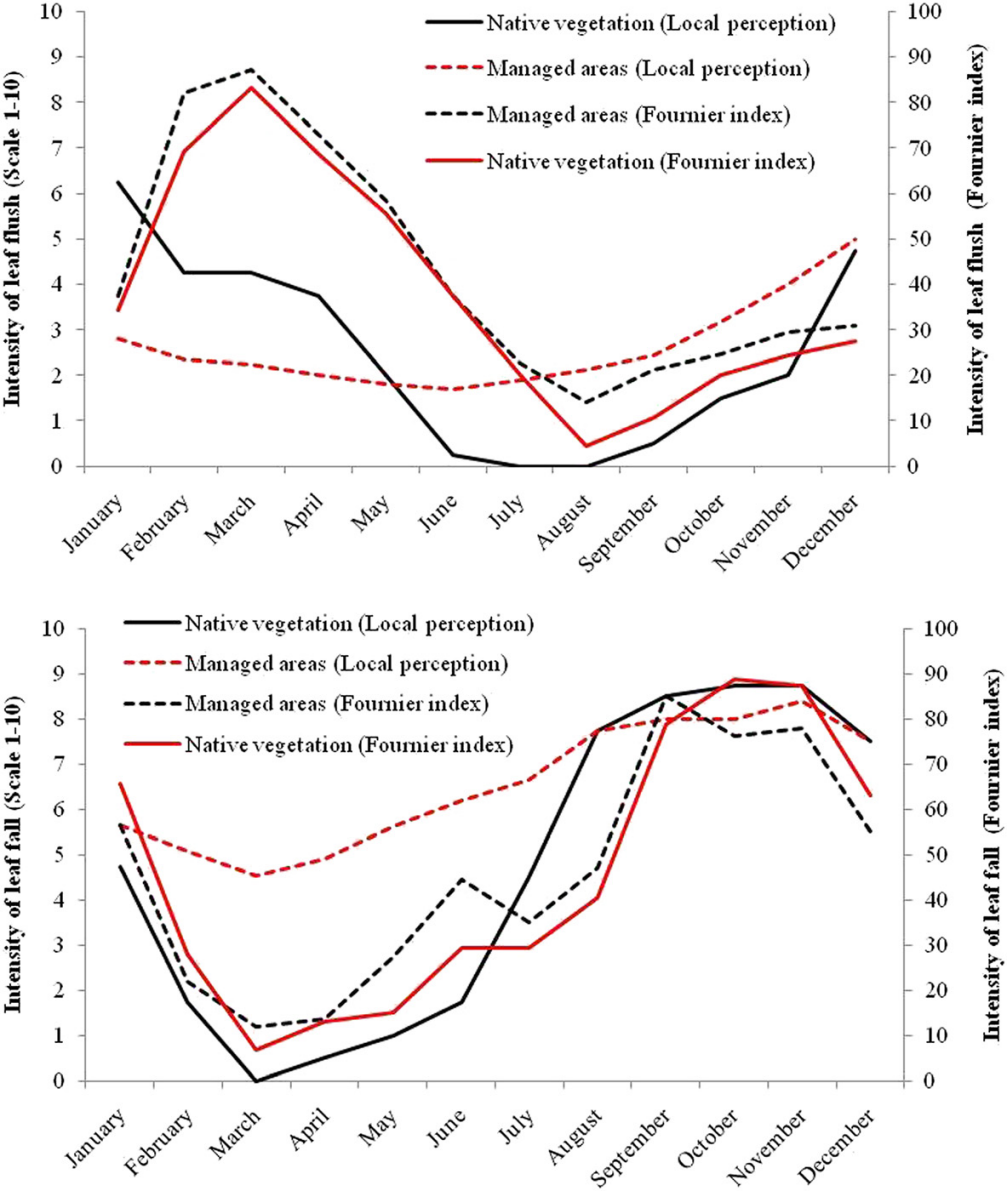
**Comparison of the intensity of leaf flush and leaf fall of *Spondias tuberosa *Arruda in managed areas (Cultivated Areas, Pasture and Homegardens) and native vegetation (Mountain and Mountain Base) with the intensity of phenophases obtained in the collaborative activity "Phenological Calendar" by members of the Carão community, city of Altinho, Pernambuco, northeastern Brazil.**

To assess significant differences between the intensity of *S. tuberosa* phenophases perceived by people in phenological activity calendar with the average intensity of phenophases of the species over two years of phenological monitoring, there has been generated a single calendar from the four built, with mean intensities perceived by the informants. Thus, we have found that as for areas of native vegetation no significant differences between the perceived intensity and average intensity of the umbuzeiro phenophases exist (flowering, fruiting F = 0.05, F = 0.002; fall leaf: F = 0.01; and leaf flush : F = 2.53, P > 0.05) (Figures 6 and 7). Similarly, there was no significant differences between the intensity perceived and the intensity monitored over two years for flowering phenophases (F = 1.9, P > 0.05) and fruiting (F = 1.81, P > 0.05) of individuals in the areas managed, however there were significant differences when it comes to foliage fall (F = 5:39, P < 0.05) and budding (F = 4.64, P < 0.05).

The local perceptions of flowering and fruiting periods for native and managed areas were very similar to data observed in the field, particularly in relation to the phenophase peaks (Table 2). The onset of fruiting varied from November to January in native vegetation and from October to January in managed areas; only one group mentioned March as the onset of fruiting (Table 2). The final fruiting ranged from June to July, with one group indicating May as the end of fruiting (Table 2). The mean duration of the phenophase also varied between areas, with the native vegetation showing a mean duration of six months and managed areas showing a mean duration of seven months. However, as noted earlier, in the areas of native vegetation fruiting began and ended later in relation to managed areas. The fruiting periods observed over the two years of monitoring were well matched with the information obtained from the local people. These data show the similarity with the information from the Phenological Calendar. The fruiting peak was the variable that resulted in the best match between the two data collection strategies (Table 2).

**Table 2:** **Comparative table of the peak periods of occurrence and the phenophases of *Spondias tuberosa *Arruda (Anacardiaceae), obtained through the collaborative approach "Phenological Calendar" (former to 4 groups with around 6 person/group) and the phenological monitoring in the field in the city of Altinho, Pernambuco, northeastern Brazil**

			**Flowering**		**Fruiting**		**Leaf flush**		**Leaf fall**	
**Phenological Calendar**			**Period**	**Peak**	**Period**	**Peak**	**Period**	**Peak**	**Period**	**Peak**
Group 1	Native		Dec–Mar	Jan	Mar–Jun	Apr	Jan–Jun	Apr	Jul–Feb	Oct–Dec
	Managed		Nov–Feb	Dec	Jan–Jun	Mar	Jan–May	Mar	May–Feb	Aug–Nov
Group 2	Native		Oct-Mar	Dec	Nov–Jul	Apr	Oct–Dec	Dec	Apr–Jan	Aug–Sep
	Managed		Oct–Mar	Dec	Oct–Jun	Mar	Oct–Dec	Dec	Apr–Jan	Aug–Sep
Group 3	Native		Oct–Mar	Jan	Jan–May	Mar	Sep–Apr	Jan–Feb	Jun–Jan	Oct-Nov
	Managed		Oct–Mar	Jan	Jan–May	Mar	Oct–Mar	Dec–Jan	Jun–Jan	Sep–Nov
Group 4	Native		Dec–May	Feb	Dec–Jun	Apr	Dec–Jan	Dec	Aug–Jan	Dec
	Managed		Nov–Apr	Jan	Dec–Jun	Mar	Jan	Jan	Aug–Feb	Nov–Dec
**Phenological monitoring**										
1st year	Native	Mountain	Sep–Feb	Nov–Dez	Nov–May	Mar	Oct–Jun	Feb	Jan–Dec	Nov
		Mountain Base	Sep–Feb	Nov	Nov–Apr	Mar	Jan–Dec	Mar	Jan–Dec	Nov
	Managed	Cultivation	Aug–Apr	Nov	Nov-Jun	Mar	Jan–Dec	Mar	Jan–Dec	Nov
		Pasture	Aug–Mar	Nov	Nov–May	Mar	Jan–Dec	Feb–Mar	Jan–Dec	Nov
		Homegardens	Aug–Feb	Nov	Nov–May	Feb–Mar	Jan–Dec	Mar	Jan–Dec	Nov
2nd year	Native	Mountain	Oct–Mar	Dec	Nov–Jul	Mar	Jan–Dec	Mar–Apr	Jul–Feb	Oct–Jan
		Mountain Base	Sep–Mar	Nov	Nov–Jun	Mar	Jan–Dec	Mar–Apr	May–Feb	Sep–Oct
	Managed	Cultivation	Sep–Mar	Nov	Nov-Jun	Mar	Jan–Dec	Feb–Apr	May–Feb	Sep–Oct
		Pasture	Sep–Mar	Nov	Nov–Jul	Jan–Mar	Jan–Dec	Feb–May	May–Feb	Sep–Oct
		Homegardens	Sep–Mar	Nov	Nov–Jun	Mar	Jan–Dec	Feb–Apr	Jan–Dec	Sep–Oct

Leaf flush was the phenophase that showed the greatest difference between the local people’s perception and the monitoring data, especially concerning the duration of the phase. However, the peak leaf flush was very similar between the Phenological Calendar and the monitoring data (Table 2). The perception of leaf fall throughout the year was similar among groups, mainly because they do not recognize differences in leaf fall between native vegetation and managed areas. Compared with field data, there were differences in the period of occurrence of this phenophase, but there was some consensus about its duration (Table 2). In areas of native and managed vegetation, the same average length of the phenophase was recorded (eleven months), whereas local people’s perceptions indicated a mean of eight months for areas of native vegetation and nine months for managed areas (Table 2). The peak of this phenophase was the variable of greatest similarity when comparing the two methodological approaches (Table 2).

Finally, people who participated in the activity, when asked about aspects that would influence the phenology behaviour of the species, were categorical in pointing out the rains during the dry season as the only variable that can negatively affect the flowering and therefore the fruiting of the species.

## Discussion

### Phenological pattern and comparison between managed and native vegetation areas

The intensity of all phenophases, as well as the timing of these events were similar between the landscape units, suggesting that human management seems not to have changed significantly the phenological pattern of species. We are going to discuss some ecological aspects that may explain this uniformity, as well as the discussion of the phenological variation of individuals in managed and unmanaged areas.

The phenological results of flowering (occurring in the dry season) and fruiting (late dry season and early rainy season) obtained here are similar to those reported in studies with *S. tuberosa* in other Caatinga areas [32-34]. Because they only occur once a year and last a few weeks, the phenological patterns of flowering and fruiting of *S. tuberosa* can be classified as cornucopia, according to Gentry [52], or annual, according to Newstrom et al. [53].

The flowering and leaf flush of *S. tuberosa* observed during the dry season, as observed in other Caatinga plants [14,54,55] can be explained by the presence of organs that can accumulate water (such as roots) [56] and by the stem, which has a low wood density [18]. The phenology of plants that accumulate water does not depend on rainfall [57,58], even in ecosystems such as arid environments where rainfall strongly influences the phenology [14,18,54,59]. It is believed that some species of tropical dry forest plants flourish in the dry season because the wet season is used for vegetative growth [60,61]. Moreover, the typical loss of leaves during the dry season favors flower visibility for pollinators [60].

Although flowering and fruiting may vary within and among populations [62], individuals of the same species that occupy adjacent regions tend to have similar phenological events during similar periods due to phylogenetic constraints [39]. This explains the similarity in synchronization and intensity of phenological events of *S. tuberosa* among the LUs. The similarity in phenological patterns observed among areas under different management regimes has been reported by Oaxaca-Villa et al. [43] in wild and managed populations of *Escontria chiotilla* (Cactaceae) in a semi-arid region of Mexico. However, other studies emphasize that in managed areas the intensity of fruit is higher when compared to unmanaged areas [23,24,27]. Individuals of cultivated populations of *Stenocereus stellatus*, species in an advanced state of domestication, have presented an increased number of flowers in anthesis if compared to individuals of wilderness areas [24]. Barreto et al. [32], while studying populations of *Spondias tuberosa* recorded more flowers and less fruits in natural areas in relation to managed areas. They attributed these differences to the fact that there is major pollinator activity (and therefore less competition for pollinators among individual plants) in managed areas, resulting in higher chances of fruit formation. Another study conducted with populations of *S. tuberosa* has also found that individuals located in corn fields, areas under intensive soil management, produced significantly more inflorescences if compared to other landscape units studied [9]. However, these authors found that there is no significant difference in the fruit production of the species if considered the landscape units studied, in addition to that, despite the intense flowering, reproductive success was limited to only one fruit per inflorescence in most individuals. The authors conclude that the possible explanation is the sharing of pollinators with individuals of other species that flower during the same period, such as *Myracrodruon urundeuva* Fr All (Anacardiaceae), *Prosopis juliflora* (Sw.) DC. and *Mimosa tenuiflora* Bent. (Fabaceae - Mimosoideae), making the reproductive success so low. Accordingly, the present results, at first sight, suggest that populations of *S. tuberosa* are not reflecting in their phenological behavior, mainly, in flowering and fruiting, the changes related to environmental changes, however, when considering the findings of Almeida et al. [9], seems to be clear that ecological interactions maintained by the species indicate a new structure for communities, suggesting an indirect effect of the domestication of landscapes.

As for phenophases overlap, the strong synchrony among individuals of *S. tuberosa* within and among the LUs supports the idea that in dry tropical forests there is a high seasonal synchrony in phenological patterns [57]. Phenological patterns possibly result from selective pressure exerted either by abiotic factors (such as intensity and duration of the dry season) [63,64] or by biotic factors (including interactions with animals such as herbivores, pollinators and seed dispersers) [65,66]. Thus, the synchrony observed here is especially important for flowering, since it favors gene flow among individuals and thus contributes to the maintenance of genetic variability in populations [19]. A study conducted in isolated populations of *Spondias mombin* in Panama also recorded a high synchrony in flowering and fruiting [11]; this synchrony may therefore be a characteristic of the genus.

These observations allow us to conclude that beyond the truism that species respond differently to the effects of handling, due to genetic and environmental heterogeneity [41], the strong synchrony observed between individuals located in different management areas indicate that timing mechanisms as for reproductive isolation have not occurred [24]. Probably this explains what we have observed in this study. This finding reinforces that an interspecific sharing of pollinators is the most likely explanation for the low reproductive success observed in populations of *S. tuberosa* under different soil management ways [9]. On the other hand, populations of *Polaskia chichipe* has its flowering peak ranging among wild populations, cultivated and managed ones, and being later in the latter [23]. This observed pattern provides reproductive isolation of populations, since individuals within populations are visited by pollinators at different times of individuals from other populations [23].

### Phenological calendar versus phenological monitoring

The similarity between phenological patterns recorded in the field and those obtained from the Phenological Calendar (especially in relation to flowering and fruiting) reveal how traditional knowledge may be useful for understanding biological phenomena. The greatest similarity in relation to reproductive phenology may be explained by the fact that *S. tuberosa* occupies a prominent place in the local community, with well-known characteristics and food uses (of fruits) [38]. Moreover, the species provides shade for animals during the dry season in the Caatinga by maintaining its leaves, which reinforces the importance of *S. tuberosa* for the local population [38]. Nevertheless, the leaf flush phase had the lowest similarity with the phenological monitoring data.

In the Yucatán peninsula, a study conducted with *Spondias pupurea* also found that people are familiar with the phenophases of this kind, highlighting the flowering and fruiting, showing the existence of a clear relationship between the local classification of fruit types with the phenology of *Spondias pupurea*[28]. For example, people recognize and classify *S. purpurea* in three main classes, which are consistent with the period of the year in which the fetching of the fruit is done. However, this study advances if related to the latter establishing a direct comparison of the perception of activity and intensity of *S. tuberosa* phenophases with concrete data as for the species phenology monitoring. Thus, the phenological calendar proposed here reveals itself as an innovative and efficient concerning the access to knowledge on biological phenomena.

Another interesting aspect reported by Ruenes-Morales et al. [28] is the influence of rain on the productivity of *S. purpurea* individuals. According to the authors, informants reported that when the rains come during the dry season they affect the annual harvest, reducing fruit production. This perception was also evidenced by Carão people, who have also pointed the rain as the environmental variable that would affect the production of umbuzeiro individuals.

The aspects presented above, together with the studies conducted by Lins Neto et al. [38] and Lins Neto et al. [45] reinforce the prominence of *S. tuberosa* in Carão community, highlighting the close relationship people have with this plant. This way, our study provides strong evidences that the popular knowledge accumulated over generations is an excellent tool for rapid diagnosis of the phenology of a plant species. Phenological studies require long periods of observation to generate robust data and to increase predictive power [29,53,67,68]. However, when decisions must be made quickly and long-term studies are infeasible, the use of traditional knowledge may facilitate appropriate and immediate conservation strategies.

Studies carried out in different part of the world, be concluded phenological studies satisfactory indicators of environmental changes, especially changes in ecosystem processes [69-72]. Thus, the perception of variations and reflections of these phenological changes within a global perspective reveals itself as importatnt tool for rapid diagnosis environment, assisting in the construction and development of strategies for the conservation of natural resources. However, it should be noted that the Phenological Calendar does not replace conventional phenological analysis, but complements it, especially for constructing future projections. This is because local people have knowledge of climate cycles that are not always detected in short term phenological studies, as they may occur at time intervals that do not coincide with the research period. Most importantly, more studies need to be developed in order to confirm the usefulness of traditional knowledge in the inference of plant phenology, especially reproductive phenophases, for other species and other ecosystems. Therefore, other systems and other approaches must be considered for strengthen the proposal that traditional knowledge is strong enough for diagnoses of phenology as confident indicator for several purposes such as climate change and conservation.

## Competing interests

The authors declare that they have no competing interests.

## Authors’ contributions

All authors contributed with writing of the manuscript. All authors read and approved the final manuscript.
